# Polymycovirus Infection Sensitizes *Aspergillus fumigatus* for Antifungal Effects of Nikkomycin Z

**DOI:** 10.3390/v15010197

**Published:** 2023-01-10

**Authors:** Gabriele Sass, Ioly Kotta-Loizou, Marife Martinez, David J. Larwood, David A. Stevens

**Affiliations:** 1 California Institute for Medical Research, San Jose, CA 95128, USA; 2 Department of Life Sciences, Faculty of Natural Sciences, Imperial College London, South Kensington Campus, London SW7 2AZ, UK; 3 Department of Clinical, Pharmaceutical and Biological Science, School of Life and Medical Sciences, University of Hertfordshire, College Lane Campus, Hatfield AL10 9AB, UK; 4 Valley Fever Solutions, Tucson, AZ 85719, USA; 5 Division of Infectious Diseases and Geographic Medicine, Department of Medicine, Stanford University School of Medicine, Stanford, CA 94305, USA

**Keywords:** *Aspergillus fumigatus*, polymycovirus, nikkomycin Z, therapy, biofilm, antifungal drugs

## Abstract

Infection with Aspergillus fumigatus polymycovirus 1 (AfuPmV-1) weakens resistance of *Aspergillus fumigatus* common reference strain Af293 biofilms in intermicrobial competition with *Pseudomonas aeruginosa*. We compared the sensitivity of two infected and one virus-free Af293 strains to antifungal drugs. All three were comparably sensitive to drugs affecting fungal membranes (voriconazole, amphotericin) or cell wall glucan synthesis (micafungin, caspofungin). In contrast, forming biofilms of virus-free Af293 were much more resistant than AfuPmV-1-infected Af293 to nikkomycin Z (NikZ), a drug inhibiting chitin synthase. The IC50 for NikZ on biofilms was between 3.8 and 7.5 µg/mL for virus-free Af293 and 0.94–1.88 µg/mL for infected strains. The IC50 for the virus-free *A. fumigatus* strain 10AF was ~2 µg/mL in most experiments. NikZ also modestly affected the planktonic growth of infected Af293 more than the virus-free strain (MIC 50%, 2 and 4 µg/mL, respectively). Virus-free Af293 biofilm showed increased metabolism, and fungus growing as biofilm or planktonically showed increased growth compared to infected; these differences do not explain the resistance of the virus-free fungus to NikZ. In summary, AfuPmV-1 infection sensitized *A. fumigatus* to NikZ, but did not affect response to drugs commonly used against *A. fumigatus* infection. Virus infection had a greater effect on NikZ inhibition of biofilm than planktonic growth.

## 1. Introduction

Virus infections are widespread in the fungal kingdom, and since the 1970s, many *Aspergillus* wild-type strains have been naturally infected [1]. Aspects of virus infection of *Aspergillus*, including effects on fungal physiology and virulence, have been recently reviewed and detailed [1,2]. Among the viruses infecting *Aspergillus* is the *Polymycoviridae* family, which was initially reported in 2015 [3], and received an official ICTV virus taxonomy profile in 2022 [4]. The *Polymycoviridae* family currently accommodates one genus, *Polymycovirus*, with 10 species, including *Aspergillus fumigatus polymycovirus. Polymycoviridae* and related viruses usually have four to eleven [5,6] dsRNA genomic segments. The majority of polymycoviruses are not conventionally encapsidated [3,5], although filamentous particles have been reported in one case [7], and are infectious as dsRNA [3,7,8]. Little is known about how viral infection affects *Aspergillus* physiology or virulence. Where effects on fungal physiology have been delineated, the results have shown either enhancing or deleterious effects [1,2].

Af293 is a very common and fully sequenced *A. fumigatus* laboratory strain [9], naturally infected with Aspergillus fumigatus polymycovirus 1 (AfuPmV-1) [3]. Using naturally infected, cured (virus-free), and (AfuPmV-1)-reinfected versions of Af293 [3], we recently found that culture filtrates from *P. aeruginosa* inhibited infected Af293 to a greater extent than virus-free Af293, dominantly shown in the face of iron stress in competition with *P. aeruginosa* [10]. The virus effect on the competition with iron was shown to relate to the timing of the production of internal and external siderophores by the fungus [11]. The same study also showed decreased susceptibility of virus-free fungus to *P. aeruginosa* volatiles [10], where the mechanism of action is unrelated to iron stress but related to the production of small organic molecules by *P. aeruginosa* [12]. Recently we studied another stress, that of high salt concentrations, where the response in the virus-infected strains was also impaired, compared to the isogenic virus-free strain [13].

Here, we compared the effects of the conventional antifungal drugs amphotericin B (AmB), voriconazole (VCZ), micafungin (MICA), and caspofungin (CASPO), as well as the chitin synthase inhibitor nikkomycin Z (NikZ) on isogenic-infected or virus-free fungus.

## 2. Materials and Methods

**Materials**: RPMI 1640 medium, 2,3-bis(2-methoxy-4-nitro-5-sulfophenyl)-2H-tetrazolium-5-carboxanilide inner salt (XTT), and menadione were purchased from Sigma-Aldrich (St. Louis, MO, USA). Nikkomycin Z (NikZ) powder was donated by Valley Fever Solutions, Tucson. Voriconazole (VCZ; Pfizer, New York City) was prepared in DMSO. Further dilutions were prepared in RPMI. Final DMSO concentrations were below 1% (i.e., 0.001%), which does not affect *A. fumigatus* biofilm metabolism, allowing omission of separate DMSO controls. Amphotericin B (AmB) was obtained from Gilead Sciences (Foster City, CA, USA), caspofungin (CASPO) was obtained from Merck (Readington Township, NJ, USA), and micafungin (MICA) was obtained from Fujisawa Pharmaceutical Co., Ltd. (Osaka, Japan). Large batches of all drugs were frozen as aliquots. For each experiment, a fresh aliquot was used.

**Strains, isolates, and institutional review:** Table 1 details *A. fumigatus* isolates used in this study. The use of all strains mentioned in Table 1 is approved by the CIMR Biological Use Committee (approval no. 001-03Yr.17).

The naturally infected Af293 strains 10–53 and 18–95 were kept in separate laboratories (10–53 in the US, 18–95 in the UK) for over 10 years. Strains 18–95 were cured from AfuPmV-1 using the protein synthesis inhibitor cycloheximide [3], resulting in strains 18–42, 18–42B, and 18–42C. While strains 18–42, 18–42B, and 18–42C were different strains derived similarly from the same curing process of 18–95. Strains 18–95B and 18–95C are separate clones of 18–95, each verified as containing the virus. Purified AfuPmV-1 was re-introduced in the virus-free *Aspergillus* 18–42 by protoplast transfection [3], producing the reinfected strain used here, 19–40. The presence or absence of AfuPmV-1 was confirmed by Northern blotting and RT-qPCR as previously described [3].

**Assays used to quantify fungal growth of biofilm or planktonic cultures**:

Biofilm: We placed 100 µL/well of a 10^5^ conidia/mL suspension in RPMI per strain and assay into a 96-well plate with five replicates. Plates were incubated overnight at 35 °C before determining optical density at 562 nm using a plate reader (Vmax, Molecular Devices, San Jose, CA, USA). Background (determined by the absorbance at 562 nm by 100 µL RPMI per well, incubated on the same plate) was subtracted. Absorbance of the virus-free 18–42 strains was regarded as 100%, and absorbance of infected strains was put in relation to that.

Planktonic growth: One milliliter of a 10^5^ conidia/mL suspension in RPMI per strain and assay was placed into 4 mL tubes. Tubes were incubated for 48 h at 35 °C before determining transmission at 530 nm, using a spectrophotometer (Genesys 20, Thermo Fisher Scientific Inc., Waltham, MA, USA). The background was subtracted by normalizing to RPMI (transmission of 100%). Fungal growth was determined by the formula: growth = 100 − T530. Growth of the virus-free strains 18–42 was regarded as 100%, and growth of infected strains was put in relation to that.

*Aspergillus* forming biofilm metabolism assay: *A. fumigatus* conidia (2.5 × 10^5^ conidia/mL, final concentration) were distributed into the wells of 96-well plates in a volume of 50 µL/well. Drug dilutions were added in 50 µL/well to the final concentrations indicated. Final volumes in wells during assays were 100 µL. Effect of RPMI 1640 medium on fungus served as the negative control. The assay plates were incubated at 37 °C overnight, and hyphal growth was verified by optical microscopy before performing XTT assays.

All experiments were evaluated by XTT metabolic assay as detailed previously [16,17]. Briefly, 150 µL of an XTT/menadione mixture (150 µg/mL XTT, 30 µM menadione) was added to each test well and incubated without agitation at 37 °C for one hour. Measurement of biofilm metabolism was performed using a plate reader (Vmax, Molecular Devices, San Jose, CA, USA) at 490 nm, following transfer of 100 µL liquid contents from each well to a fresh 96-well plate.

Determination of 50% antifungal activity (IC50): Figures and Figure Legends indicate drug dilutions in RPMI, in 1:2 dilution steps, that encompassed a concentration that was closest to inhibiting 50% of fungal metabolism and is referred to as the IC50 of a drug on a certain isolate.

Minimal inhibitory concentration (MIC), minimal fungicidal concentration (MFC): One hundred microliters of NikZ were distributed into tubes. Then, 900 µL of standardized inoculum was added to the tubes to achieve final concentrations of NikZ 0.5–128 µg/mL. Tubes were incubated for 48 h at 35 °C before reading MICs. Planktonic growth and inhibition in an MIC assay were evaluated as previously described [18,19]. MIC 50% was defined as the lowest drug dilution allowing half as much fungal growth as seen in tubes not containing drug. MIC 95% was defined as the lowest drug dilution, where only traces of fungal growth were visible. MIC 100% was defined as the lowest drug dilution where no fungal growth was visible. To determine the MFC, 50 µL of each tube with traces of fungal growth or without visual growth was plated on Sabouraud agar and incubated at 35 °C for 24 h. MFC was considered the minimal concentration of the drug resulting in killing ≥99% of the inoculum.

**Statistics:** Results were analyzed using Student’s *t*-test if two groups were compared, and 1-way ANOVA combined with a Tukey’s post-test for multiple comparisons. All data in this study are expressed as a mean ± SD. Data are also reported as the percent of control. Each assay was performed with three to four biological and four to twelve technical replicates. Representative experiments are shown.

## 3. Results

### 3.1. NikZ Dose-Dependently Interfered with Forming Biofilm Metabolism of Two A. fumigatus Reference Strains and Caused Structural Changes in Hyphae

**Figure 1:**
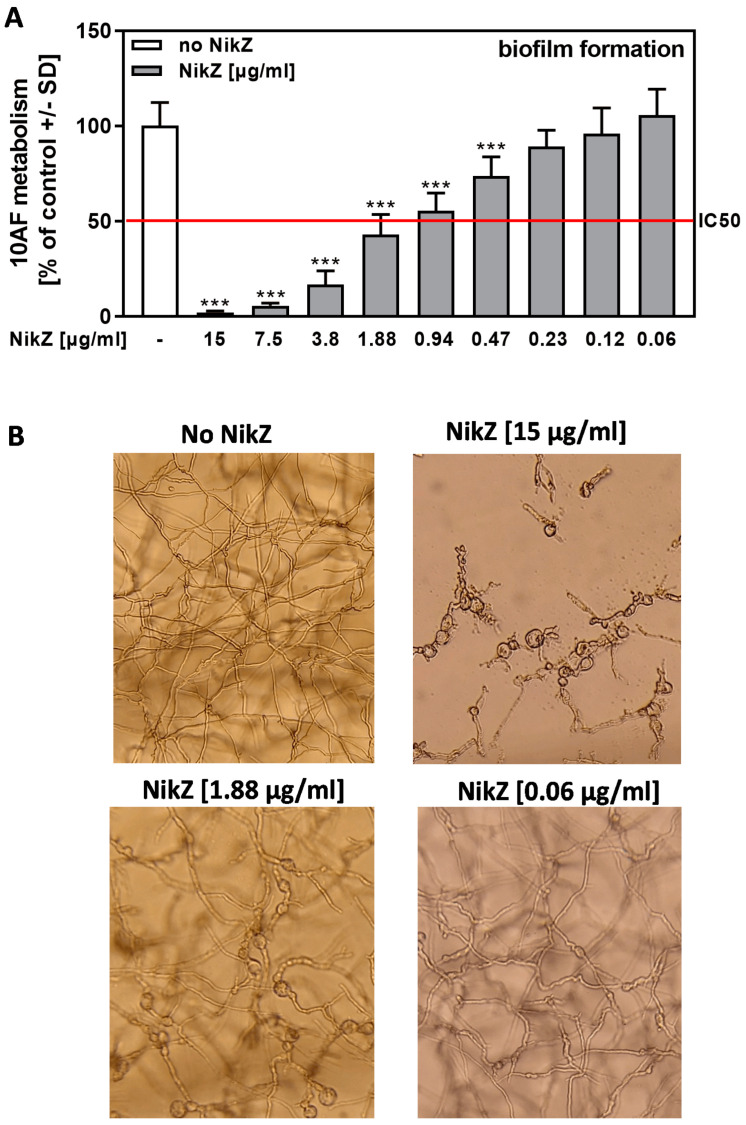
NikZ dose-dependently interferes with 10AF, forming biofilm metabolism and causes structural changes in hyphae. (**A**) The 10AF forming biofilm was incubated with NikZ (15 to 0.06 µg/mL) at 37 °C for 16 h. *Aspergillus* metabolism was measured by XTT assay. Metabolism in the presence of RPMI alone (white bar) was regarded as 100%. The red line represents the IC50. Comparisons: No NikZ (white bar) vs. each NikZ concentration (grey bars). Statistical analysis (n = 6): Unpaired *t*-test, three asterisks = *p* ≤ 0.001. Only significant differences are indicated. (**B**) Pictures were taken from wells described in A after 16 h of incubation at 37 °C before XTT analysis. A representative picture is shown. All pictures were taken at 125× magnification.

NikZ significantly interfered with the metabolism of forming biofilms of 10AF at concentrations > 0.47 µg/mL, with the IC50 between 0.94 and 1.88 µg/mL in most experiments (Figure 1A). Microscopic evaluation showed that NikZ, at the concentrations tested, interfered with fungal growth as well, but did not prevent hyphal formation completely, even at the highest concentration used, 15 µg/mL (Figure 1B). NikZ caused the formation of balloon-like or knob-like structures in hyphae that were not observed in untreated controls (Figure 1B). These structures, at a lesser size and frequency, occurred at NikZ concentrations that had no significant effects on fungal metabolism, i.e., NikZ 0.06 µg/mL (compare Figure 1A to Figure 1B).

**Figure 2:**
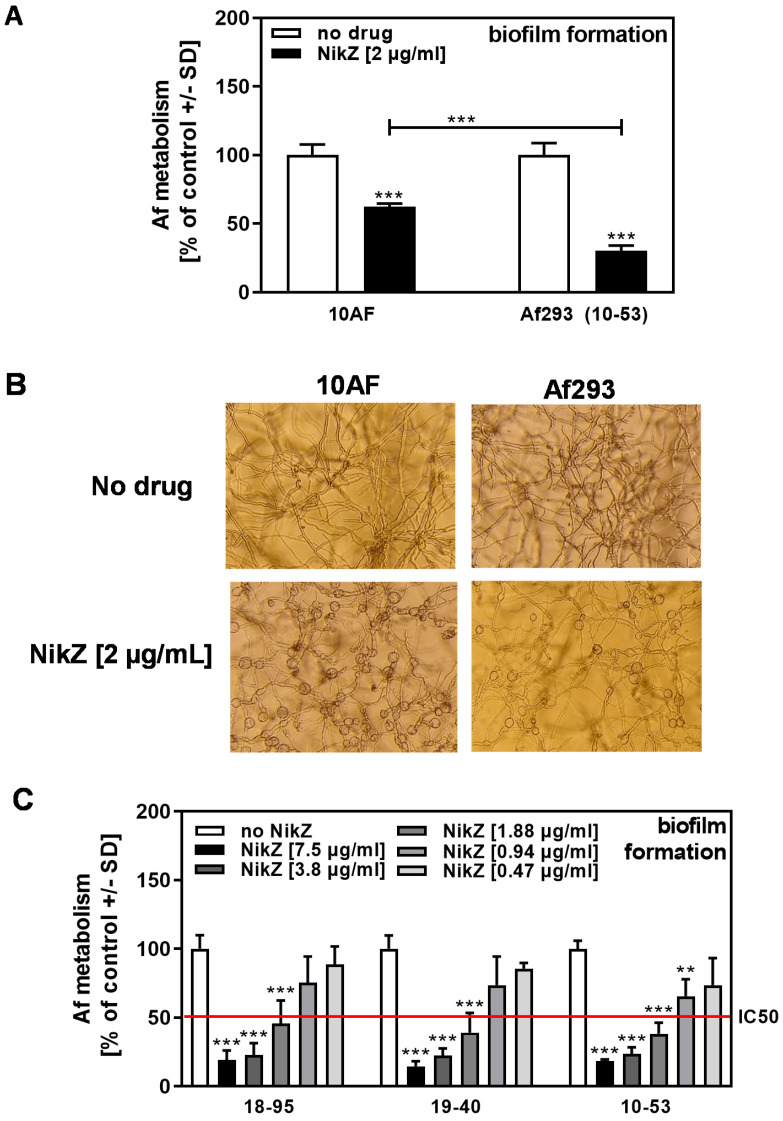
Comparison of NikZ effects among three *A. fumigatus* reference strains. (**A**) The 10AF and Af293 forming biofilm was incubated with NikZ (2 µg/mL) at 37 °C for 16 h. *Aspergillus* metabolism was measured by XTT assay. Metabolism in the presence of RPMI alone (white bars for each strain) was regarded as 100%. Comparisons: No NikZ (white bars) vs. NikZ (black and grey bars) for each strain. Other comparisons as indicated by the ends of the brackets. Statistical analysis (n = 8): Unpaired *t*-test, three asterisks = *p* ≤ 0.001. (**B**) Pictures were taken from wells described in A after 16 h of incubation at 37 °C before XTT analysis. A representative picture is shown. All pictures were taken at 125x magnification. (**C**) Infected Af293 forming biofilm (strains 18–95 (naturally infected), 19–40 (reinfected), and 10–53 (naturally infected)) was incubated with NikZ (0.47 to 7.5 µg/mL) at 37 °C for 16 h. *Aspergillus* metabolism was measured by XTT assay. Metabolism in the presence of RPMI alone (white bars for each strain) was regarded as 100%. Comparisons: No NikZ (white bars) vs. all bars containing NikZ for each strain. Statistical analysis (n = 4): 1-way ANOVA, one, two, or three asterisks = *p* ≤ 0.05, *p* ≤ 0.01, or *p* ≤ 0.001, respectively. Only significant differences are indicated.

When NikZ (2 µg/mL) effects were compared between the virus-free strain *A. fumigatus* strain 10AF and the naturally AfuPmV-1-infected *A. fumigatus* strain Af293 (10–53), we found 10–53 to be more sensitive to NikZ than 10AF (Figure 2A). Microscopic evaluation showed that, at the NikZ concentration used in Figure 2A, balloon-like structures occurred in both *A. fumigatus* strains (Figure 2B); the frequency of these structures also appears dose-related. The IC50 for NikZ against infected Af293 strains 10–53, 18–95, and 19–40 was determined to be between 0.94 and 1.88 µg/mL (Figure 2C).

### 3.2. Infected Af293 Is More Sensitive to NikZ Than Virus-Free Af293

**Figure 3:**
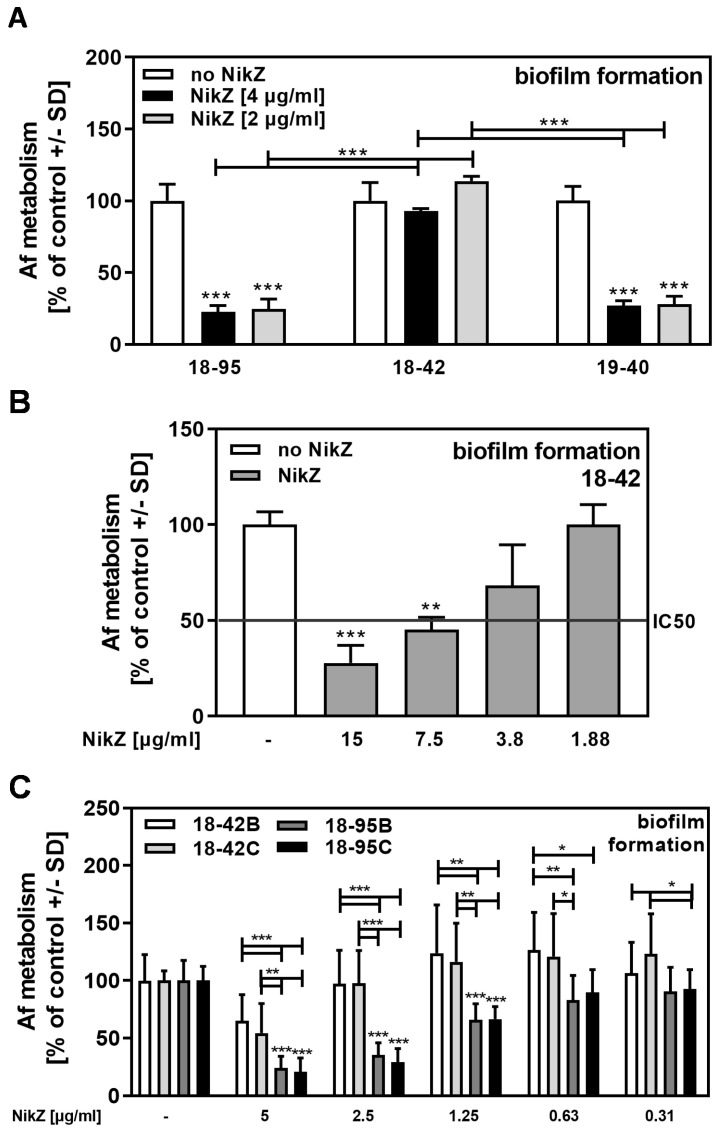
Infected Af293 is more sensitive to NikZ than virus-free Af293. (**A**) Infected (18–95, 19–40) or virus-free (18–42) *Aspergillus*-forming biofilm was incubated with NikZ (2 or 4 µg/mL) at 37 °C for 16 h. Fungal metabolism was measured by XTT assay. Metabolism in the presence of RPMI alone (white bars for each strain) was regarded as 100%. Comparisons: No NikZ (white bars) vs. NikZ (black and grey bars) for each strain. Other comparisons are indicated by the ends of the brackets. Statistical analysis (n = 4): Unpaired *t*-test, three asterisks = *p* ≤ 0.001. Only significant differences are indicated. (**B**) Strains 18–42 forming biofilm were incubated with NikZ (15 to 0.06 µg/mL) at 37 °C for 16 h. Fungal metabolism was measured by XTT assay. Metabolism in the presence of RPMI alone (white bar) was regarded as 100%. The red line represents the IC50. Comparisons: No NikZ (white bar) vs. each NikZ concentration (grey bars). Statistical analysis (n = 4): Unpaired *t*-test, two and three asterisks = *p* ≤ 0.01 and *p* ≤ 0.001, respectively. Only significant differences are indicated. Concentrations 0.94 or 0.06 µg/mL also did not demonstrate inhibition and are not shown here to simplify presentation. (**C**) Infected (18–95B, 18–95C) or virus-free (18–42B, 18–42C) *Aspergillus*-forming biofilm was incubated with NikZ (0.31 or 5 µg/mL) at 37 °C for 16 h. Fungal metabolism was measured by XTT assay. Metabolism in the presence of RPMI alone (leftmost bars) was regarded as 100%. Comparisons: No NikZ vs. all NikZ concentrations for each strain. Other comparisons as indicated by the ends of the brackets. Statistical analysis (n = 8): 1-way ANOVA, one, two, or three asterisks = *p* ≤ 0.05, *p* ≤ 0.01, or *p* ≤ 0.001, respectively. Only significant differences are indicated.

We next compared three Af293-based strains for their sensitivity to NikZ, of which two strains (18–95 and 19–40) were infected, and a third strain, 18–42, was virus-free (see Section 2 for details). Our results show that both infected strains were significantly more sensitive to NikZ than the virus-free strain (Figure 3A). A dose-response curve for NikZ on 18–42 revealed the IC50 to be between 3.8 and 7.5 ug/mL (Figure 3B), which is 2 to 4 times higher than the IC50 on infected Af293 strains (compare Figure 3B to Figure 2C). In addition to 18–42, we used two more cured, virus-free isolates, 18–42B and C, which, like 18–42, were obtained separately by curing 18–95 from AfuPmV-1 infection. These virus-free isolates, like 18–42, were significantly more resistant to NikZ than 18–95, with an IC50 ≥ 5 µg/mL (Figure 3C).

### 3.3. AfuPmV-1 Infection Does Not Sensitize A. fumigatus Biofilm to Conventional Drugs

**Figure 4:**
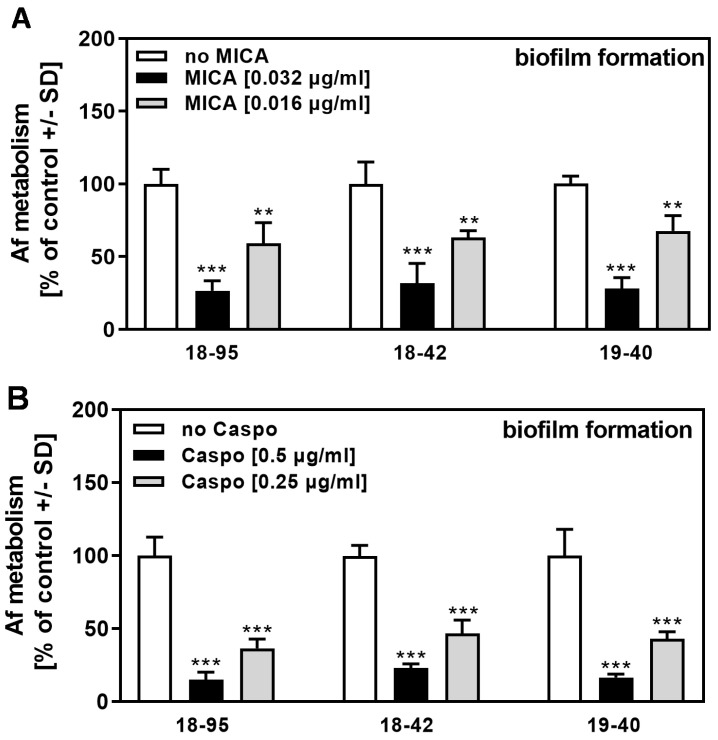
AfuPmV-1 infection does not sensitize *A. fumigatus* to MICA or CASPO. Infected (18–95, 19–40) or virus-free (18–42) *Aspergillus*-forming biofilm was incubated with (**A**) MICA (0.016 or 0.032 µg/mL) or (**B**) CASPO (0.25 or 0.5 µg/mL) at 37 °C for 16 h. Fungal metabolism was measured by XTT assay. Metabolism in the presence of RPMI alone (white bars for each strain) was regarded as 100%. Comparisons: No drug (white bars) vs. drug (black and grey bars) for each strain. Statistical analysis (n = 4): Unpaired *t*-test, two or three asterisks = *p* ≤ 0.01, or *p* ≤ 0.001, respectively. No significant differences in drug response were observed when comparing infected strains (18–95 and 19–40) to the virus-free strain (18–42).

**Figure 5:**
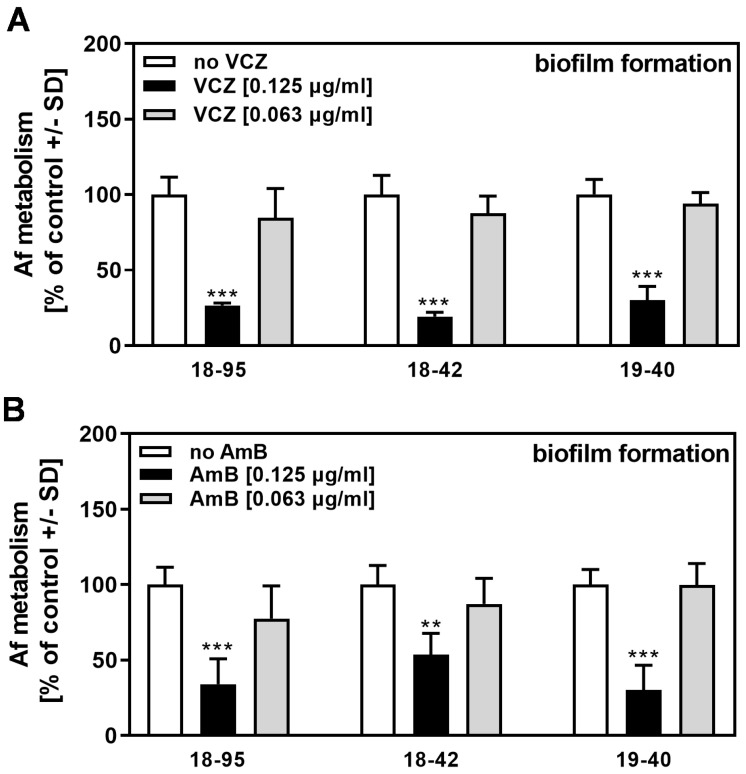
AfuPmV-1 infection does not sensitize *A. fumigatus* to VCZ or AmB. Infected (18–95, 19–40) or virus-free (18–42) *Aspergillus* forming biofilm was incubated with (**A**) VCZ (0.063 or 0.125 µg/mL), or (**B**) AmB (0.063 or 0.125 µg/mL) at 37 °C for 16 h. Fungal metabolism was measured by XTT assay. Metabolism in the presence of RPMI alone (white bars for each strain) was regarded as 100%. Comparisons: No drug (white bars) vs. drug (black and grey bars) for each strain. Statistical analysis (n = 4): Unpaired *t*-test. Two or three asterisks = *p* ≤ 0.01 or *p* ≤ 0.001, respectively. No significant differences in drug response were observed when comparing infected strains (18–95 and 19–40) to the virus-free strain (18–42).

In assays similar to those used to determine the NikZ effects shown in Figure 3A, we examined the effects of four drugs conventionally used for the treatment of *A. fumigatus* infections. Virus infection of *A. fumigatus* did not increase its sensitivity to MICA (Figure 4A), CASPO (Figure 4B), VCZ (Figure 5A), or AmB (Figure 5B). These results for conventional drugs were similar to those obtained for planktonic fungal growth in a recent study by our group [10].

### 3.4. Virus-Free Planktonic Af293 Is Less Sensitive to NikZ Than Infected Fungus

We now were interested in how NikZ affected AfuPmV-1-infected compared to virus-free Af293. Our results showed that NikZ effects on virus-free Af293 (strain 18–42) were weaker than on infected strains (Table 2). With respect to the inhibition of 50% of growth (MIC 50%), we found a two-fold difference between infected and virus-free strains. With respect to 95% inhibition of growth (MIC 95%, only traces of fungus visible), the difference was at least 3- to 4-fold. Total inhibition of visible growth (MIC 100%), as well as sterilization of the inoculum (MFC) for all strains, was higher than the highest tested NikZ concentration of 128 µg/mL (Table 2).

### 3.5. Increased Growth or Metabolism Does Not Explain Decreased Sensitivity of Virus-Free Aspergillus to NikZ

**Figure 6:**
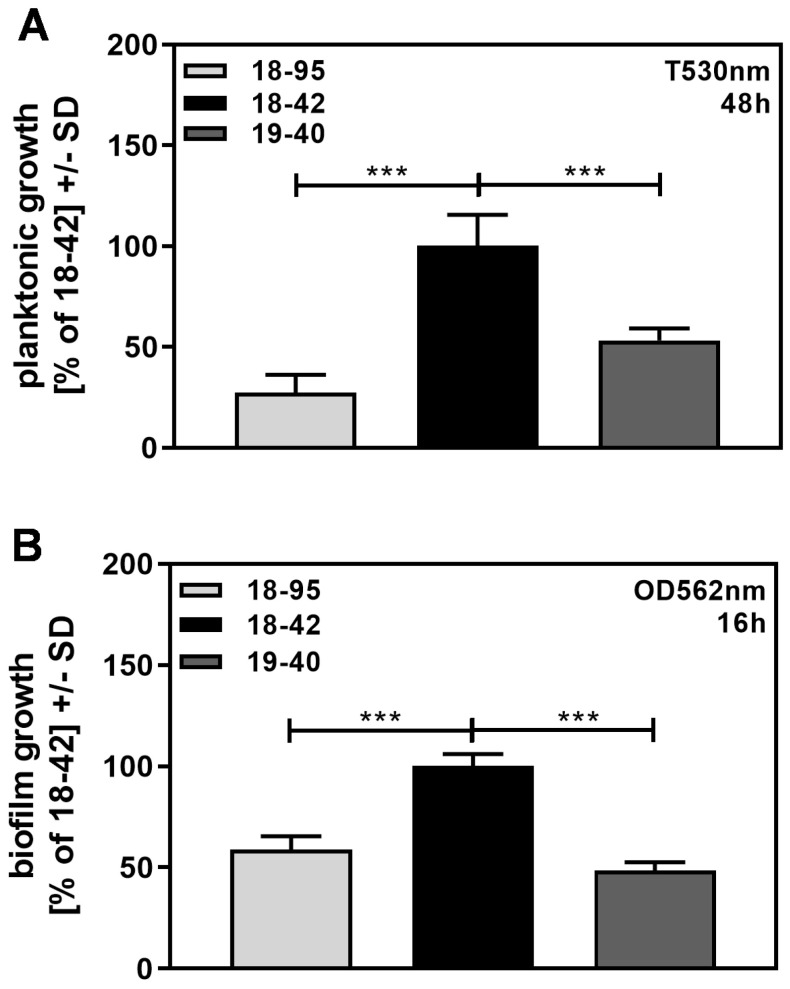
Virus-free *A. fumigatus* grows more than infected fungus. Infected (18–95, 19–40) or virus-free (18–42) *Aspergillus* was grown either planktonically in tubes for 48 h (**A**) or forming a biofilm in a 96-well plate for 16 h (**B**). After incubation, fungal growth was determined photometrically, as described in the Section 2. Growth of 18–42 was regarded as 100%. Comparisons: as indicated by the ends of the brackets. Statistical analysis (A, n = 6; B, n = 5): Unpaired *t*-test. Three asterisks = *p* ≤ 0.001.

We observed that virus-free Af293, growing planktonically (Figure 6A), as well as in the form of biofilm (Figure 6B), grew significantly better than infected Af293.

**Figure 7:**
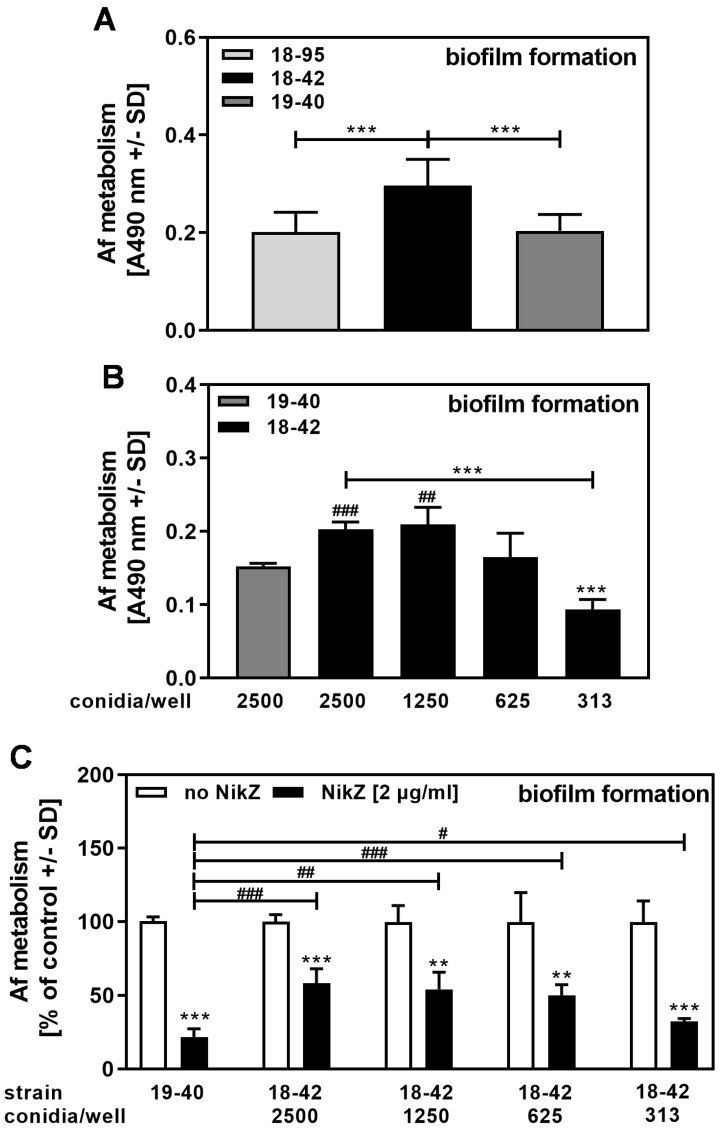
Increased growth does not explain decreased sensitivity of virus-free *Aspergillus* to NikZ. (**A**) Infected (18–95, 19–40) or virus-free (18–42) *Aspergillus*-forming biofilm was incubated at 37 °C for 16 h. Fungal metabolism was measured by XTT assay and is presented as A490 nm. Comparisons as indicated by the ends of the brackets. Statistical analysis (n = 12): Unpaired *t*-test. Three asterisks = *p* ≤ 0.001. (**B**) Infected (19–40) or virus-free (18–42) *Aspergillus*-forming biofilm was used, starting from 2500 per well. For 18–42, two-fold dilution steps were added. The assay was incubated at 37 °C for 16 h. Fungal metabolism was measured by XTT assay and is presented as A490 nm. Comparisons: 19–40 (grey bar, 2500 conidia per well) vs. 18–42 (black bars, conidia dilutions), or as indicated by the ends of the bracket. Statistical analysis (n = 4): Unpaired *t*-test. Two or three asterisks or pound signs = *p* ≤ 0.01, *p* ≤ 0.001, respectively. Only significant differences are indicated. Asterisks represent decreases, pound signs represent increases in fungal metabolism. (**C**) Infected (19–40, 2500 conidia/well) or virus-free (18–42, 2500 conidia per well, and 2-fold dilution steps) *Aspergillus*-forming biofilm was incubated with NikZ (2 µg/mL). The assay was incubated at 37 °C for 16 h. Fungal metabolism was measured by XTT assay. Metabolism in the presence of RPMI alone (white bars for each pair) was regarded as 100%. Comparisons: RPMI (white bar) vs. NikZ (black bars) for each pair, or as indicated by the ends of the bracket. Statistical analysis (n = 4): unpaired *t*-test. One, two, or three asterisks or pound signs = *p* ≤ 0.05, *p* ≤ 0.01, *p* ≤ 0.001, respectively. Asterisks represent decreases, pound signs represent increases in fungal metabolism.

Similarly, biofilm assays showed that virus-free fungus had significantly more metabolism than infected fungus (Figure 7A). We considered whether its increased ability to grow rendered virus-free fungus less sensitive to NikZ.

We measured biofilm metabolism in 18–42 cultures that started with the same amount of conidia as used in Figure 7A, 2500 conidia per well, diluted stepwise to as little as 1/8 of that concentration, 313 conidia per well (Figure 7B). At increasing starting conidial concentrations, increased levels of metabolic activity were measured at the end of the assay. Comparing metabolism between these conidia concentrations for 18–42 to metabolism of 19–40 (2500 conidia/well), we found similar metabolism between 19–40 (2500 conidia/well) and 18–42 (625 conidia/well) (Figure 7B). When NikZ (2 µg/mL) was assessed with these two conidial concentrations, and metabolism of the control for each conidial concentration was defined as 100%, it became clear that even when normalized to equal metabolism (625 conidia/well in 18–42; 2500 in 19–40), virus-free fungus was still more resistant to NikZ (Figure 7C).

This study showed that even when we compensated for the differences in basal metabolism between virus-free and infected, NikZ effects on infected strains were still greater. Thus, the increased basal metabolism of the virus-free strain does not explain the decreased NikZ effect on it.

## 4. Discussion

Virus infection of *Aspergillus* is not a rare event. There are multiple virus families with members that are known to infect *Aspergillus*, encompassing *Chrysoviridae*, *Narnaviridae*, *Partitiviridae*, *Totiviridae,* and *Polymycoviridae* [20]. The infection rate by mycoviruses among *Aspergillus* species has been described to be between 10 and 80% [20,21], while between 7 and 19% of clinical *A. fumigatus* isolates are infected [22,23]. Infection with mycoviruses seems to be latent, persistent, and difficult to eliminate [20,24].

The clinical relevance of *Aspergillus* virus infection is controversial. There are reports showing hypovirulence in a mouse model [25], increased virulence [1,26,27], or no change in virulence whether infected or not infected [1,27,28]. In two studies using planktonic fungal growth, no altered susceptibility towards conventional antifungal drugs has been detected [10,28].

We also found that virus-free and mycovirus-infected *A. fumigatus* strains, in the form of planktonically growing cultures, but also in the form of biofilms, were comparably sensitive to conventional drugs affecting fungal membranes (voriconazole, amphotericin B), or cell wall glucan synthesis (micafungin, caspofungin). In contrast, forming biofilms as well as planktonic cultures of virus-free Af293 were much more resistant than AfuPmV-1-infected Af293 to nikkomycin Z (NikZ), a drug inhibiting chitin synthase. The IC50 for NikZ on biofilms was between 3.8 and 7.5 µg/mL for virus-free Af293 and between 0.94 and 1.88 µg/mL for infected isogenic Af293 strains. The IC50 for the virus-free *A. fumigatus* strain 10AF was approximately 2 µg/mL in most experiments. NikZ also modestly affected the planktonic growth of AfuPmV-1-infected Af293 more than it affected the virus-free strain (MIC 50% virus-free 4 µg/mL, infected 2 µg/mL). Virus-free Af293 biofilm showed increased metabolism, and when growing in the form of biofilm, or planktonically, showed increased growth compared to infected Af293.

Our results indicate that virus infection of *Aspergillus* renders the fungus more susceptible to stresses affecting cell wall formation, especially towards NikZ, affecting chitin synthase. It would be of interest to compare the effects of other chitin synthase inhibitors, e.g., [29], to NikZ effects on infected vs. virus-free fungus in future studies. Stress induced by other inhibitors of cell wall synthesis (echinocandins) is not amplified by viral infection, indicating specificity for the kind of cell wall stress.

We observed that virus-free fungus grew better compared to infected fungus, suggesting that viral infection might impair cell wall synthesis, particularly at specific points in the synthesis of the wall. Although we could show that resistance of virus-free biofilm to NikZ was not caused by its better growth, we indicate that virus-altered cell wall synthesis is more prone to detrimental effects caused by inhibition of chitin synthesis. This inhibition might result in osmotic stress for the fungal cell, resulting from impaired cell wall synthesis, and either lead to reduced growth or cell injury.

In contrast to our data, infection of a plant pathogenic fungus, *Penicillium digitatum*, with a polymycovirus resulted in increased susceptibility to an azole drug [8]. Although *Penicillium* and *Aspergillus* are both filamentous fungi, the higher susceptibility of infected *Penicillium* to azole drugs might be explained by differences in cell wall stress response [30].

We previously showed that virus infection of *A. fumigatus* alters responses to a variety of stresses, such as iron deficiency [10,11], *Pseudomonas* volatiles [10], or high salt [13]. Whether virus infection renders *A. fumigatus* more susceptible to other stresses is currently under investigation. Higher susceptibility of infected strains to a variety of stresses points to the virus affecting molecules that have been proposed as master regulators of stress responses or cell wall stress responses [31,32,33]. It might also be possible that under stress conditions, virus accumulation would increase and further increase host sensitivity, e.g., to NikZ. Further studies need to investigate these hypotheses.

## Figures and Tables

**Table 1:** *A. fumigatus* strains used in this study.

Strain	ATCC Number	CIMR Number	Origin	AfuPmV-1 Infection	Ref.
10AF	90240		Virulent patient isolate	Not infected	[14,15]
Af293	MYA-4609	10–53	USA	Naturally infected	[9]
Af293	MYA-4609	18–95	UK	Naturally infected	[9]
Af293	MYA-4609	18–95B	UK	Naturally infected	[9]
Af293	MYA-4609	18–95C	UK	Naturally infected	[9]
Af293		18–42	UK	18–95 cured	[3]
Af293		18–42B	UK	18–95 cured	[3]
Af293		18–42C	UK	18–95 cured	[3]
Af293		19–40	UK	18–42 reinfected	[3]

**Table 2:** Effects of NikZ on planktonic fungal growth at 48 h of incubation. Concentrations (µg/mL). The same results were obtained in two independent experiments.

Strain	MIC 50%	MIC 95%	MIC 100%	MFC
18–95	2	8	>128	>128
18–42	4	>128	>128	>128
19–40	2	16	>128	>128
10–53	2	8	>128	>128

## Data Availability

Data supporting the reported results are available from the corresponding author.
